# Supplementary material for: Serological Cross-Reactions between Expressed VP2 Proteins from Different Bluetongue Virus Serotypes
Source: Viruses. 2021 Jul 26;13(8):1455. doi: 10.3390/v13081455 (PMC8402635; doi:10.3390/v13081455)
Supplement: Supplementary file 1 [file viruses-13-01455-s001.zip › viruses-1269507-si.pdf]

Serological Cross-Reactions between Expressed VP2 Proteins from Different Bluetongue Virus Serotypes

Petra C. Fay [1,2], Fauziah Mohd Jaafar [3], Carrie Batten [2], Houssam Attoui [3], Keith Saunders [4], George P. Lomonossoff [4], Elizabeth Reid [1], Daniel Horton [5], Sushila Maan [6], David Haig [1], Janet M. Daly and Peter P. C. Mertens [1,]

Supplementary Data:

Figure S1 (supplementary): Neighbour-joining phylogenetic tree for the carboxy and amino terminal halves of BTV VP2

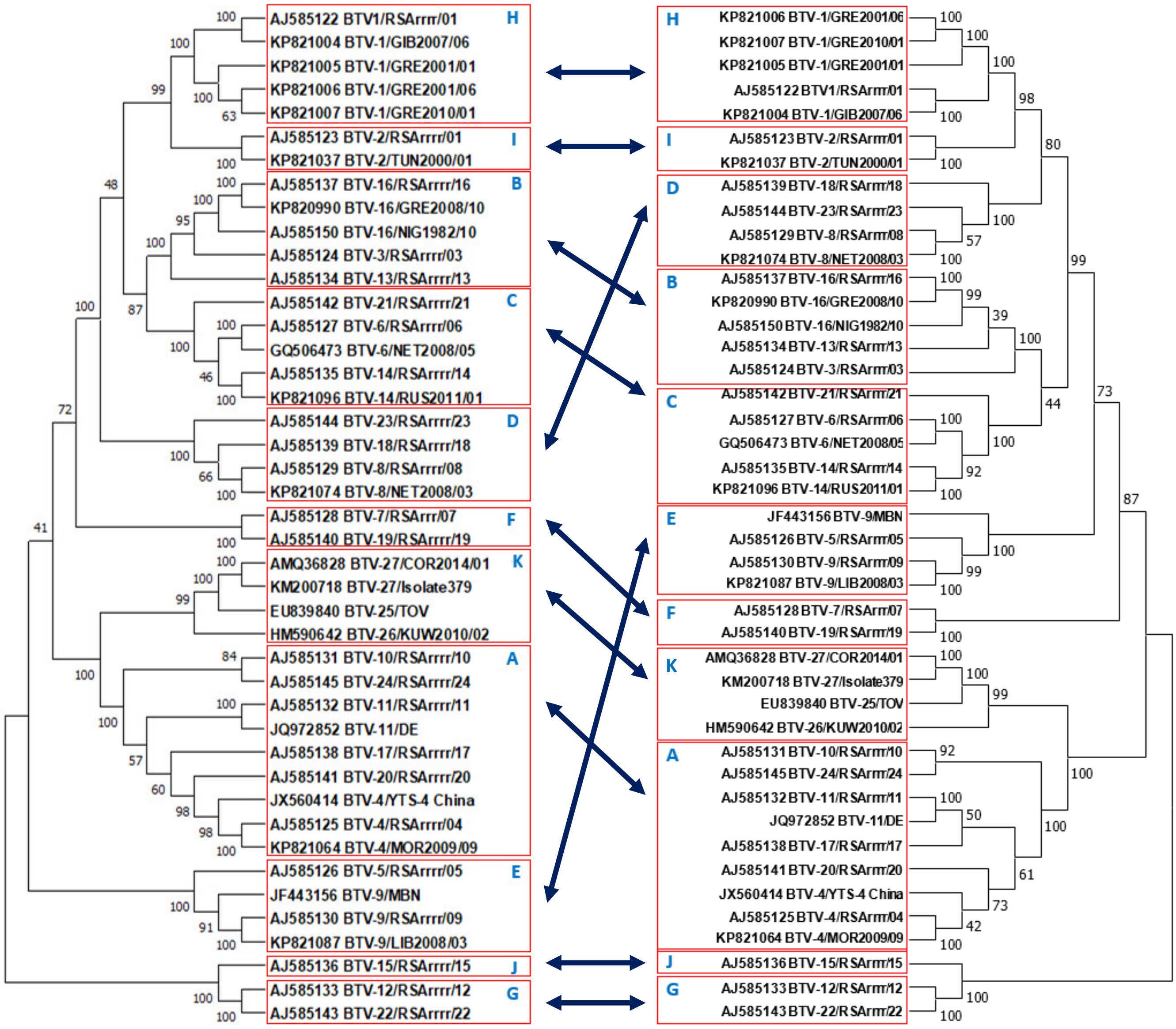

Figure S1 (supplementary): A neighbour-joining phylogenetic tree constructed with aa sequences of VP2(OC1) of BTV-1 to BTV-27 depicting phylogenetic groupings. The N-terminal domain of the VP2 (aa 1-480, relative to VP2 sequence of BTV-1/RSArrrr/01: AJ585122) is shown on the left part of the figure, while the C-terminal domain of the VP2 (aa 481-961, relative to VP2 sequence of BTV-1/RSArrrr/01: AJ585122) is shown on the right. The trees were generated using the p-distance algorithm (pairwise deletion) implemented in the MEGA X software programme. The arrows indicate the relative positions of the different VP2 nuceotype groups in the two trees. Neither sub-domain tree is identical to that constructed for the whole protein sequence (Figure 1).

Table S1 (supplementary). Antigenic map optimisation outputs for rabbit anti-BTV-rVP2 and sheep anti-BTV reference-sera by I-ELISA and SNT.

Map Dimensions	Rabbit anti-BTV-rVP2 sera				Sheep anti-BTV reference-sera			
	I-ELISA		SNT		I-ELISA		SNT	
	**Sum-Squared error**	**Table vs Map Distance Correlation (r^2)**	**Sum-Squared error**	**Table vs Map Distance Correlation (r^2)**	**Sum-Squared error**	**Table vs Map Distance Correlation (r^2)**	**Sum-Squared error**	**Table vs Map Distance Correlation (r^2)**
2D	202.7745	0.82	1.6698	0.99	191.6525	0.83	6.0857	0.99
3D	174.1195	0.87	1.6698	0.99	159.7894	0.88	6.0857	0.99
4D	173.2870	0.87	1.6698	0.99	151.5304	0.89	6.0857	0.99
5D	173.1036	0.88	1.6698	0.99	149.1482	0.90	6.0857	0.99

Maps were optimised 500 times each.

Table S1 (supplementary): Endpoint I-ELISA and SNT titres for the rabbit anti-BTV-rVP2 and sheep post BTV-infection sera were modelled using the ACMACS software (https://acmacs-wieb.antigenic-cartography.org/) to determine optimum map dimensions that best represent the antigenic relationships observed. Optimised map dimensions that generated the lowest sum-squared error and the highest table vs map distance correlation coefficient (r_2) were selected for analysis (indicative of optimal map positioning achieved for each data point). On this basis 3D maps were selected to best represent the multidimensional nature of the antigenic relationships between the different serotypes.

Table S2 (Supplementary): Antigenic distance (units) calculated from I-ELISA data, for expressed rVP2 proteins, using rabbit anti-rVP2 sera

BTV	α-rVP2	AU	bAb titre
1W	S1w	0	40960
	S1e	1	20480
	S4w	0	40960
	S6w	1	2560
	S8w	6	640
	S11w	2	10240
	S14w	5	1280
	S25	2	1280
	S26	3	1280
	S27	2	2560
1E	S1w	0	40960
	S1e	0	40960
	S4w	7	320
	S6w	0	5120
	S8w	5	1280
	S11w	5	1280
	S25	3	640
	S26	4	640
	S27	2	2560
2W	S1w	2	10240
	S4w	9	80
	S6w	5	160
	S8w	10	40
	S11w	5	1280
	S14w	10	40
	S25	7	40
	S26	8	40
	S27	4	640
4W	S1w	5	1280
	S4w	0	40960
	S6w	1	2560
	S8w	5	1280
	S11w	0	40960
	S14w	4	2560
	S25	2	1280
	S26	2	2560
	S27	1	5120

BTV	α-rVP2	AU	bAb titre
4E	S1w	5	1280
	S4w	3	5120
	S6w	1	2560
	S8w	7	320
	S11w	2	10240
	S14w	7	320
	S25	0	5120
	S26	1	5120
	S27	0	10240
10w	S1w	5	1280
	S4w	4	2560
	S6w	1	2560
	S8w	5	1280
	S11w	4	2560
	S14w	7	320
	S25	1	2560
	S26	2	2560
	S27	0	10240
11w	S1w	5	1280
	S4w	1	20480
	S6w	2	1280
	S8w	8	160
	S11w	0	40960
	S14w	6	640
	S25	2	1280
	S26	2	2560
	S27	0	10240
6w	S1w	6	640
	S4w	6	640
	S6w	1	2560
	S8w	8	160
	S11w	5	1280
	S14w	8	160
	S25	4	320
	S26	5	320
	S27	2	2560

BTV	α-rVP2	AU	bAb titre
14w	S1w	2	10240
	S6w	0	5120
	S8w	5	1280
	S11w	4	2560
	S14w	0	40960
	S25	1	2560
	S26	2	2560
	S27	0	102403
8w	S6w	3	640
	S8w	0	40960
	S11w	8	160
	S27	6	160
9w	S1w	5	1280
	S4w	5	1280
	S6w	1	2560
	S8w	7	320
	S11w	4	2560
	S14w	7	320
	S25	1	2560
	S26	2	2560
	S27	0	10240
9e	S1w	4	2560
	S4w	3	5120
	S6w	0	5120
	S8w	4	2560
	S11w	3	5120
	S14w	6	640
	S25	1	2560
	S26	1	5120
	S27	1	5120
16w	S1w	5	1280
	S4w	5	1280
	S6w	0	5120
	S8w	6	640
	S11w	4	2560
	S14w	7	320
	S25	1	2560
	S26	1	5120

BTV	α-rVP2	AU	bAb titre
16w	S27	1	5120
16e	S1w	5	1280
	S4w	7	320
	S6w	0	5120
	S8w	6	640
	S11w	3	5120
	S14w	5	1280
	S25	2	1280
	S26	2	2560
	S27	2	2560
25	S1w	9	80
	S4w	6	640
	S6w	1	2560
	S8w	10	40
	S11w	5	1280
	S25	3	640
	S26	4	640
	S27	1	5120
26	S1w	4	2560
	S1e	10	40
	S4w	3	5120
	S6w	0	5120
	S8w	4	2560
	S11w	2	10240
	S14w	5	1280
	S25	1	2560
	S26	1	5120
	S27	0	10240
27	S1w	4	2560
	S4w	4	2560
	S6w	0	5120
	S8w	5	1280
	S11w	3	5120
	S14w	5	1280
	S25	1	2560
	S26	0	10240
	S27	0	10240

Table S2 (Supplementary): Shows the antigenic distances for the rabbit anti-rVP2 sera, calculated from I-ELISA data using the expressed rVP2 proteins as target antigens. The expressed VP2 proteins were derived using sequence data from more recent BTV strains from Europe, the Mediterranean reagion and other selected geographic locations (distinct topotypes) and the anti-rVP2 sera generated were therefore derived the same strains of BTV. Text colour coding matches the coding used for different nucleotypes in figures 2, 3 and 4 and table S2 to S5.

Table S3 (Supplementary): Antigenic distance (antigenic units) calculated from I-ELISA data for expressed rVP2 proteins, using sheep anti-BTV reference-sera

BTV	Ref sera	AU	bAb titre
1W	S1W	0	5120
	S2W	10	10
	S5W	5	10
	S6W	0	5120
	S10W	4	320
	S16e	6	40
	S17w	4	20
	S20e	7	10
1E	S1W	2	1280
	S2W	6	160
	S3W	2	20
	S5W	1	160
	S6W	9	10
	S8w	6	40
	S10w	2	1280
	S11w	3	40
	S12w	2	20
	S13w	0	10
	S16e	5	80
	S17w	3	40
	S19w	4	20
	S20e	5	40
	S21e	2	10
	S24w	4	10
	S26e	7	10
2W	S2W	0	10240
	S5W	4	20
	S10w	2	1280
	S16e	6	40
	S17w	1	160
	S19w	5	10
	S20w	5	40
4w	S2W	7	80
	S3w	3	10
	S4w	0	10240
	S5w	2	80
	S8w	5	80
	S9w	4	640
	S11w	2	80
	S16e	3	329
	S17w	0	320
	S18w	3	10
	S19w	5	10
	S20e	0	1280
	S21e	2	10
	S22w	0	10
	S24w	1	80
4E	S2W	8	40
	S5W	3	40
	S8w	7	20
	S10w	4	320
	S11w	4	20
	S13w	0	10
	S16e	6	40
	S17w	2	80
	S19w	5	10
	S20e	6	20
10w	S2W	6	160
	S5W	1	160
	S8w	5	80
	S10w	2	1280
	S11w	1	160
	S16e	3	320
	S17w	0	320
	S19w	2	80
	S20e	4	80
	S26e	5	40
11w	S2W	9	20
	S5W	4	20
	S10w	4	320
	S11w	3	40
	S16e	6	40
	S17w	3	40
	S19w	4	20
6w	S1W	7	40
	S2W	0	10240
	S3W	0	80
	S5W	0	320
	S6W	8	20
	S8w	2	640
	S10w	1	2560
	S11w	0	320
	S12w	1	40
	S16e	3	320
	S17w	2	80
	S19w	3	40
	S20e	4	80
14w	S2W	6	160
	S5W	2	80
	S10w	5	160
	S11w	5	10
	S14w	0	1280
	S15w	0	160
	S17w	2	80
	S21e	0	40
8w	S2W	7	80
	S5W	2	80
	S8w	0	2560
	S10w	2	1280
	S11w	3	40
	S16e	4	160
	S17w	4	20
	S19w	4	20
	S20e	5	40
9w	S2W	9	20
	S5W	3	40
	S8w	7	20
	S9w	1	5120
	S10w	3	640
	S11w	5	10
	S12w	1	40
	S16e	6	40
	S17w	3	40
	S17w	0	80
	S19w	0	320
	S20e	6	30
	S21e	0	40
	S23e	0	160
	S24w	0	160
	S26e	0	1280
9e	S2W	8	40
	S5W	2	80
	S8w	6	40
	S9w	0	10240
	S10w	2	1280
	S11w	4	20
	S12w	0	80
	S13w	0	10
	S16e	6	40
	S17w	3	40
	S20e	6	20
	S21e	2	10
16w	S2W	7	80
	S5W	4	20
	S8w	4	160
	S10w	4	320
	S16e	4	160
	S17w	1	160
	S20e	6	20
16e	S2w	8	40
	S5W	1	160
	S8w	3	320
	S10w	0	5120
	S16e	0	2560
	S17w	0	320
	S20e	5	40
25	S2W	6	160
	S3W	2	20
	S5W	0	320
	S8w	2	640
	S10w	3	640
	S11w	2	80
	S12w	0	80
	S16e	5	80
	S17w	2	80
	S19w	4	20
	S20e	6	20
26	S2W	6	160
	S5W	2	80
	S8w	5	80
	S10w	2	1280
	S11w	5	10
	S16e	6	40
	S17w	4	20
	S19w	5	10
	S20e	6	20
	S26e	7	10
27	S2W	9	20
	S5W	4	20
	S10w	4	320
	S11w	4	20
	S16e	6	40
	S17w	2	80
	S18w	3	10
	S19w	4	20
	S20e	5	40
	S21e	1	20
	S23e	4	10
	S24w	4	10

Table S3 (Supplementary): Shows the antigenic distances for the sheep reference antisera, calculated from I-ELISA data using the rVP2 proteins. The expressed VP2 proteins were not derived from the same reference strains of BTV but were generated using sequence data from more recent BTV strains from Europe, the Mediterraneam reagion and other selected geographic locations (distinct topotypes). Text colour coding matches the coding used for different nucleotypes in figures 2, 3 and 4 and table S2 to S5.
